# Manganese Accumulation in the Brain via Various Transporters and Its Neurotoxicity Mechanisms

**DOI:** 10.3390/molecules25245880

**Published:** 2020-12-12

**Authors:** Ivan Nyarko-Danquah, Edward Pajarillo, Alexis Digman, Karam F. A. Soliman, Michael Aschner, Eunsook Lee

**Affiliations:** 1 Division of Pharmaceutical Sciences, Florida A&M University, Tallahassee, FL 32307, USA; ivan1.nyarkodanquah@famu.edu (I.N.-D.); edward.pajarillo@famu.edu (E.P.); alexis1.digman@famu.edu (A.D.); karam.soliman@famu.edu (K.F.A.S.); 2 Department of Molecular Pharmacology, Albert Einstein College of Medicine Bronx, New York, NY 10461, USA; michael.aschner@einsteinmed.org; 3 Laboratory of Molecular Nutrition of the Institute for Personalized Medicine, I. M. Sechenov First Moscow State Medical University, 119146 Moscow, Russia

**Keywords:** manganese, DMT1, ZIP4, ZIP8, oxidative stress, inflammation, dopamine, acetylcholine, glutamate, GABA, α-synuclein

## Abstract

Manganese (Mn) is an essential trace element, serving as a cofactor for several key enzymes, such as glutamine synthetase, arginase, pyruvate decarboxylase, and mitochondrial superoxide dismutase. However, its chronic overexposure can result in a neurological disorder referred to as manganism, presenting symptoms similar to those inherent to Parkinson’s disease. The pathological symptoms of Mn-induced toxicity are well-known, but the underlying mechanisms of Mn transport to the brain and cellular toxicity leading to Mn’s neurotoxicity are not completely understood. Mn’s levels in the brain are regulated by multiple transporters responsible for its uptake and efflux, and thus, dysregulation of these transporters may result in Mn accumulation in the brain, causing neurotoxicity. Its distribution and subcellular localization in the brain and associated subcellular toxicity mechanisms have also been extensively studied. This review highlights the presently known Mn transporters and their roles in Mn-induced neurotoxicity, as well as subsequent molecular and cellular dysregulation upon its intracellular uptakes, such as oxidative stress, neuroinflammation, disruption of neurotransmission, α-synuclein aggregation, and amyloidogenesis.

## 1. Introduction

Manganese (Mn) is a trace element in the body, found sufficiently in food [1], and thus, a Mn deficiency-causing pathological condition is rare [2]. Mn plays a critical role in proper bone formation, cell survival, metabolism, and antioxidant defense systems [2]. It serves as an essential cofactor of glutamine synthetase and superoxide dismutase, which are enzymes responsible for the glutamate-glutamine cycle and reactive oxygen species (ROS) scavenging, respectively [3]. Studies have also shown that iron and zinc can be replaced by Mn as a cofactor for some enzymes, maintaining proper function [4], indicating that, in the absence of iron and zinc, Mn can serve as a cofactor for some enzymes involved in critical biological activities.

Chronic exposure to excess levels of Mn is, however, a growing health-related concern, as it causes a neurological disorder referred to as manganism, presenting similar pathological symptoms to those of Parkinson’s disease (PD), including dopaminergic neuronal injury and motor dysfunction, as well as cognitive deficits [5]. Mn overexposure may result from environmental and/or occupational settings, such as mining, smelting, and agrochemical and biomedical industries [6,7]. Mn neurotoxicity is associated with Mn accumulation in various regions of the brain, including basal ganglia, frontal cortex, and cerebellum [8]. Moreover, increasing evidence reveals that Mn may also be a risk factor for several other neurodegenerative disorders, including Alzheimer’s disease (AD), dementia, and Huntington’s disease (HD), to name a few [9]. 

The entry of Mn into the body is via the inhalation of fumes, dust, and air particulates, as well as ingestion of contaminated food and water, leading to its subsequent accumulation in the brain. Inhaled Mn is absorbed in the lungs and enters the circulation, while ingested Mn is rapidly absorbed in intestinal epithelial cells via its transporters [10]. Mn levels in the body are regulated by multiple transporters, including transmembrane transporters such as divalent metal transporter 1 (DMT1) [11], transferrin and transferrin receptor (TR) [12], zinc-interacting proteins (ZIP8 and ZIP14) [13,14], calcium channels [15], citrate transporters [16], ferroportin (Fpn) [17], and SLC30A10 [18], as well as the intracellular trafficking proteins (SPCA1 and ATP13A2). Dysregulation of these transporters may lead to Mn accumulation in the brain, causing Mn-induced neurotoxicity [19].

The pathological consequences of Mn toxicity upon its accumulation in the brain are extensively investigated, but the molecular and cellular mechanisms underlying its detrimental effects remain poorly understood. Mn induces oxidative stress by the overproduction of reactive oxygen species (ROS) and neuroinflammation by elevating proinflammatory cytokines in various in vitro and in vivo animal models, indicating that oxidative stress and inflammation are critically involved in Mn-induced neurotoxicity [20,21,22,23]. Mn dysregulates multiple neurotransmitters, including dopamine (DA), glutamate, gamma aminobutyric acid (GABA), and acetylcholine (ACh) [24,25,26,27], indicating that Mn toxicity could affect multiple brain functions associated with these neurotransmission imbalances, such as motor deficits, learning, memory, and cognition. Although the direct link between Mn toxicity and PD-related Lewy bodies remains to be established, studies have shown that Mn dysregulated the function of α-synuclein (αSyn), a protein commonly found in Lewy bodies in the brain of PD patients [28]. Growing evidence also suggests that Mn is associated with AD via the overproduction and aggregation of amyloid-β (Aβ), one of AD’s pathological hallmarks. 

This review will focus on each Mn transporter’s contributing role in Mn accumulation in the brain and delineating the molecular and cellular mechanisms of Mn-induced neurotoxicity, particularly oxidative stress, inflammation, and aberrant neurotransmitter systems. Moreover, Mn’s potential contributing role to αSyn aggregation in PD and Aβ accumulation in AD will also be discussed.

## 2. Mn Transport to the Brain

Mn can be transported in and out of the brain by its transporters [9]. Multiple transporters for Mn have been identified, although the significant role of each of these transporters in Mn’s neurotoxicity is still under investigation [9,29,30].

Several carrier proteins are involved in Mn’s transport across the blood-brain barrier (BBB). Mn is transported into the brain via several importers, including DMT1, zinc-interacting protein 8 (ZIP8, encoded by *SLC39A8*) and ZIP14 (*SLC39A14*), transferrin and TR, citrate transporter, and calcium channels. Mn is transported into the brain either as Mn^2+^, Mn-citrate, or Mn^3+^-transferrin. All the transport mechanisms known to date play a part in delivering Mn to the brain, and it is unlikely that Mn influx across the BBB can be attributed to a single carrier.

### 2.1. DMT1

DMT1 belongs to the family of natural resistance-associated macrophage proteins (NRAMP) [31,32], which was identified as a homolog of NRAMP1, a protein involved in the host defense against several types of infections [33,34]. It is highly expressed in the plasma membrane and endosome [35], as well as the mitochondria of various animal cells [36,37], playing a role in mitochondrial iron and Mn acquisition [11]. Mutation of this gene in humans is associated with severe congenital hypochromic microcytic anemia and an iron overload [38,39]. While DMT1 is ubiquitously expressed, most notably in the proximal duodenum in the peripheral tissues [31,40,41], it is highly expressed in basal ganglia, including the caudate nucleus, the putamen, and the substantia nigra, which could explain the sensitivity of this region of the brain to Mn toxicity [42]. DMT1 is also known to be expressed in glial cells in the neocortex, subcortical white matter, and hippocampus [43]. 

Mn exposure in vivo induced DMT1 expression in the brain subventricular zone and the rostral migratory stream, and this may have partly contributed to Mn overload in the subventricular zone in association with Mn-induced aberrant adult neurogenesis [44]. Olfactory DMT1 also appeared to play a critical role in Mn uptake into the brain, as the nasal absorption of Mn is significantly reduced in DMT1-mutated *b/b* rats [45] and an iron deficiency increased the olfactory DMT1 levels, suggesting the importance of olfactory DMT1 in Mn transport to the brain and its modulation by the iron status [45]. Other studies showed that Mn crossed the BBB into the brain by DMT1-independent processes [46], indicating various transporters with different routes for Mn transport into the brain. Single nucleotide polymorphisms (SNPs) in DMT1 are associated with an increased incidence of PD [47,48]; however, whether these SNPs enhance Mn deposition, implying gain-of-function in transporters, has yet to be determined. 

### 2.2. ZIP8 and ZIP14

ZIP8 is a transmembrane protein encoded by the *SLC39A8* gene and expressed on the apical surface of brain capillaries, shuttling various divalent ions under normal biological conditions [49], including Mn transport to the brain [13]. ZIP8 is abundantly expressed in lung tissues in humans [50,51], and the alveolar epithelia of the lungs provide an entry route for inhaled Mn, indicating its important role in Mn transport into the body via lung tissues [52]. ZIP8 in the brain microvascular capillary endothelial cells also plays a critical role in Mn uptake into the brain [53]. ZIP8 expression is enriched at the apical side of human choroid plexus papilloma cells, which are related to the blood-cerebrospinal fluid (CSF) barrier, contributing to Mn accumulation in these cells [54]. These findings suggest that ZIP8 transporter is important in Mn transport into the brain. 

ZIP14, encoded by the *SLC39A14* gene, mediates the cellular uptake of Mn, zinc, and iron [14,55] and is highly expressed in the liver, kidney, and testis [56,57]. ZIP14 appears to prevent Mn accumulation in the brain. Mutations in this gene lead to an excessive accumulation of Mn in the brain, resulting in rapidly progressive childhood-onset parkinsonism [58]. The deletion of ZIP14-impaired Mn uptake by the liver and pancreas in *Slc39a14^−/−^* mice results in a reduction of gastrointestinal excretion of Mn and a subsequent Mn accumulation in the body, most notably in the bone and brain [59]. A zebrafish model lacking ZIP14 accumulated higher Mn levels in the brain compared to those in wild-type (WT), leading to altered locomotor activity [58]. ZIP14 is enriched on the basolateral side of choroid plexus cells, contributing to Mn transport through these cells and the subsequent excretion [54]. ZIP14 mutation caused Mn accumulation in early-onset parkinsonism dystonia patients, suggesting the important role of ZIP14 in the regulation of Mn levels in the brain to prevent Mn neurotoxicity in humans [58]. Studies also showed that Mn levels were elevated in the CSF of patients carrying an *SLC39A14* mutation, supporting the role of ZIP14 in lowering Mn levels in the brain [60].

### 2.3. Transferrin and Transferrin Receptor (TR)

Transferrin primarily mediates iron transport in blood circulation, but it can also transport Mn (mostly Mn^3+^) into the brain [16,61,62]. The TR, expressed in neurons, microglia, or astrocytes, binds and uptakes the Mn-transferrin complex into the cells [63,64]. The significance of the transferrin/TR-mediated transport of Mn to the brain remains unclear, since mice with low plasma transferrin levels could maintain normal brain Mn contents [12]. 

### 2.4. Citrate Transporter and Calcium Channels

Mn-citrate is believed to be the major form to transport Mn to the brain, as Mn-citrate has been reported as the most abundant Mn species found in Mn carriers in serum [65]. Mn-citrate crosses the BBB in a citrate transporter-dependent mechanism [16]. The monocarboxylate transporter and the organic anion transporter are also putative carriers for Mn-citrate into the brain [66]. However, the role of citrate in Mn transport via a specific type of citrate carrier in Mn accumulation in the brain remains to be established.

Mn also enters the brain through ligand-gated store-operated calcium channels, which are expressed in brain endothelial cells [15], as well as voltage-gated L-type calcium channels in dopaminergic neurons of the midbrain, which could contribute to the selective vulnerability of these neurons to Mn toxicity [67]. 

### 2.5. Fpn

Fpn is a transmembrane protein expressed in the plasma membrane and known to export intracellular iron in mammals [68,69]. It also exports intracellular Mn into the extracellular space, thus playing a protective role in Mn-induced cellular toxicity and oxidative stress [17]. Fpn is expressed in various brain cell types, including neurons, astrocytes, the BBB endothelial cells, oligodendrocytes, choroid plexus, and ependymal cells [70]. Fpn overexpression reduced Mn accumulation, resulting in attenuating Mn-induced cytotoxicity in the mouse brain [71], suggesting that Fpn plays an important role in regulating Mn levels in the brain.

### 2.6. SLC30A10

SLC30A10 is one of the 10 solute carrier family 30 (SLC30) transporters. Unlike the majority of the members in this family, which mediate zinc transport, the SLC30A10 transporter mediates Mn efflux [18,72], thus protecting against Mn-induced cellular toxicity. Mutation of the *SLC30A10* gene causes Mn overload syndrome, resulting in parkinsonism with hypermanganesemia [73,74]. It is currently the only known transporter associated with the familial form of Mn-induced parkinsonism [75]. The liver and gastrointestinal tract-specific *Slc30a10* knockout mice induced a marked elevation of Mn levels in the brain, suggesting that the damage that results from brain Mn is regulated by the activity of SLC30A10 in the liver and gastrointestinal tract [76]. 

### 2.7. Intracellular Mn Distribution and Storage

In addition to the plasma membrane transporters, some transporters expressed in the membranes of intracellular organelles regulate subcellular Mn transport and distribution. Studies suggest that lysosomes, the Golgi apparatus, endosomes, mitochondria, and the nucleus, are subcellular organelles for the accumulation of intracellular Mn [77,78,79].

The calcium uniporter sequesters intracellular Mn^2+^ in the mitochondria [80]. Since Mn^2+^ can be oxidized to a strong pro-oxidizing agent, Mn^3+^, by superoxide [81] and the mitochondrial electron transport chain, is the largest producer of superoxide in the cell [82]; it is believed that the oxidation of Mn^2+^ to Mn^3+^ damages mitochondria [81]. 

The transferrin/TR transports Mn into endosomes and mitochondria in mouse hippocampal and striatal neuronal cells [62], although DMT1 is also expressed in the endosomes, transporting Mn into the cytoplasm [62].

PARK9 (encoded by *ATP13A2*) is a P-type ATPase of unknown function [83], but mutations in the *ATP13A2* gene are associated with Kufor-Rakeb syndrome and are found also in patients with various other types of parkinsonism, causing an autosomal recessive parkinsonian syndrome [84]. Mutations in this gene also cause a syndrome of neurodegeneration with brain iron accumulation (NBIA), which is characterized by an abnormal accumulation of iron in the basal ganglia [85]. PARK9 is localized predominantly in lysosomes and is involved in the shuttling of cations across the membrane of the lysosome [84], helping sequester toxic metals such as Mn. PARK9 is highly expressed in the neurons of the substantia nigra, protecting neurons against Mn-induced neurotoxicity [83,84,86], suggesting its important role in protecting cells against Mn cytotoxicity via regulating intracellular Mn redistribution into vacuoles.

SPCA1 is a Ca/Mn ATPase expressed highly on the surface of the Golgi membrane, transporting Mn and calcium from the cytosol into the Golgi lumen [87,88]. This allows for the safe storage of calcium and export of excess Mn via the secretory pathway. The role of SPCA1 in Mn transport is important to maintain optimal Mn level, since its silencing or deficiency results in extreme sensitivity to Mn toxicity [89,90].

## 3. Mechanisms of Mn-Induced Neurotoxicity

Excessive Mn levels alter cellular functions by multiple mechanisms. In the brain, Mn can directly cause neuronal toxicity by the inhibition of mitochondrial respiration, leading to energy failure, oxidative stress [91], and excitotoxicity [92]. Mn can also induce neurotoxicity by impairing functions of glial cells such as astrocytes and microglia. Moreover, Mn toxicity continues to progress even after cessation of its exposure, suggesting that there are ongoing mechanisms of progression for Mn toxicity, such as inflammation and protein aggregation [2,93]. Here, we will focus on the mechanisms of Mn neurotoxicity, including oxidative stress, neuroinflammation, neurotransmitter dysregulation, and αSyn aggregation in various in vitro and in vivo experimental settings (Table 1).

### 3.1. Oxidative Stress

Oxidative stress is one of the major mechanisms of Mn-induced neurotoxicity. Mn accumulation in the brain exacerbates oxidative damage preferentially in the basal ganglia, including the globus pallidus and striatum. These regions are especially vulnerable to oxidative injury due to their intense oxygen consumption reflective of a high metabolic rate and significant DA content. Mn-increased ROS production leads to the activation of proapoptotic processes such as cytochrome c release from the mitochondria, activation of caspase-3, and DNA fragmentation in dopaminergic cell lines [94]. Mn-induced ROS-triggered mitochondria-dependent apoptotic activation was independent of caspase-8 [95] but dependent on protein kinase C delta (PKCδ) [95,96]. The antioxidant Trolox attenuated Mn-induced neurotoxicity and rescued dysfunctional dopaminergic transmission and Mn-induced motor coordination deficits in both in vitro and in vivo experimental settings [21,22], indicating that oxidative stress is a critical mechanism of Mn-induced neurotoxicity. 

In addition, Mn could exacerbate DA-induced oxidative damage in dopaminergic neurons, since DA itself can undergo oxidation to produce quinones and free radicals that can be neurotoxic and contribute to neurodegenerative processes in cells [97], as well as *Caenorhabditis elegans* [98]. The process of DA metabolism to homovanillic acid by monoamine oxidase and catechol-O-methyltransferase can also produce H_2_O_2_, leading to oxidative stress in the nigrostriatal system. 

Mn-induced impairment of the neuronal antioxidant system renders the brain more susceptible to Mn toxicity, contributing to altered striatal concentrations of glutathione, glutathione disulfide, ascorbic acid, malondialdehyde, and the activities of glutathione reductase and glutathione peroxidase [99,100,101]. Impairment of the antioxidant system may also play a role in developing neurodegenerative diseases by shifting the balance between the generation of ROS and its elimination. Glutathione provides the first-line endogenous agent of cellular defense against oxidative stress in both neurons and astrocytes. The inhibition of glutathione synthesis potentiates Mn-induced energetic impairment by increasing the hypoxanthine, xanthine, and uric acid levels in the striatum and brainstems of aged rats [102], implicating that mitochondrial dysfunction is closely associated with the Mn-induced impairment of antioxidant functions. 

Mn-induced ROS also oxidizes polyunsaturated fatty acids present in the cell membrane, leading to the production of several arachidonic acid peroxidation products, such as F2-isoprostanes, which is considered the most accurate measure of oxidative damage [103,104,105], increasing F2-isoprostanes levels leading to cellular damage in both primary neurons and astrocytes [106,107]. 

Oxidative stress can also damage cellular macromolecules such as nucleic acids, which are prone to irreparable damage from oxidative damage, since any form of oxidative modification can lead to genetic base mutations. Higher levels of the oxidized DNA product 8-hydroxyguanosine, as well as reduced 8-hydroxyl-2-deoxyguanosine, have been observed in the substantia nigra and CSF of PD patients [108,109], suggesting that oxidative stress leads to the oxidation of nucleobases. Importantly, Mn induced oxidative damage to thymine DNA bases in SH-SY5Y cells [23]. Taken together, these findings indicate that Mn-induced ROS production contributes to oxidative damage in DNA molecules and the subsequent genetic base mutations.

### 3.2. Inflammation

Glial cells, including astrocytes and microglia, represent a diverse class of cells in the brain, making up 50% of all cells in the central nervous system. The involvement of glia in Mn-induced neurotoxicity has received significant attention [93], since Mn activated glial cells in the brains of humans, nonhuman primates, and rodents [21,110,111,112], exerting lasting effects on the neuroinflammatory status of glial cells [113]. Activated astrocytes and microglia are often observed in the postmortem evaluation of Mn-exposed patients [112].

Astrocytes are well-established to play a critical role in Mn-induced neuroinflammation, as Mn accumulates preferentially in this cell type in the brain [114], potentially due to the abundant expression of high-capacity transporters [115]. In addition to DMT1 [7], the TRs expressed on astrocytic surfaces readily bind to Mn-transferrin, allowing them to retain Mn concentrations 10- to 50-fold greater than those in neurons and, thus, are more susceptible to Mn toxicity than other cell types [116]. Mn significantly increased astrogliosis in the basal ganglia of patients exposed to high Mn levels [117]. Mn released cytotoxic substances such as iNOS and IL-6 [118] and increased mRNA levels of various inflammatory genes in astrocytes [119]. Mn increased the expression of cyclooxygenase 2 and iNOS by the activation of NF-κB in astrocytes, leading to inflammation and neuronal apoptosis [118,120,121,122], suggesting that astrocytes could play an important role in Mn-induced neuroinflammation. Mn in low levels also potentiated lipopolysaccharide (LPS)-induced inflammation of glial cells and enhanced neurotoxicity [123] in astrocytes by increasing the production of TNF-α, IL-1β, and iNOS expression [20,118,124,125,126].

Microglia, another glial cell type acting as the major immune cells in the brain, play a critical role in Mn-induced neurotoxicity by producing proinflammatory cytokines [125], which can be exacerbated indirectly by Mn-stimulated astrocytes [127], leading to neuronal injury. Mn also induced the release of microglial-derived cytokines such as TNF-α, ILs, and interferons by activation of the NF-κB pathway in microglia [93,125,128]. Mn at low concentrations potentiated the LPS-induced microglial release of cytokines such as TNF-α and IL-1β [125,127]. The potentiating effect of Mn on TNF-α and IL-6 at the mRNA level was delayed but sustained for up to 24 h [129,130]. In our previous study, leucine-rich repeat kinase 2 (LRRK2) appears to mediate Mn-induced inflammation, since Mn increased the expression of LRRK2 and its kinase activity in microglia, leading to the enhancement of cytokine production such as TNF-α, but the genetic deletion or pharmacological inhibition of LRRK2 attenuated Mn-induced cytotoxicity in microglial cells [131]. 

### 3.3. Mn Effect on Neurotransmitters

Mn could interfere with multiple neurotransmission systems as it accumulates in various brain regions, comprising an intricate network of neurons that synthesize and release multiple neurotransmitters, including DA, glutamate, ACh, and GABA. In fact, Mn has been shown to dysregulate these neurotransmission systems. 

DA. Mn accumulation in the basal ganglia decreased DA levels in neonates [132], as well as adult rats [133], particularly in the dopaminergic neurons of the substantia nigra pars compacta, where dopaminergic cell bodies are located [77]. In addition, Mn also decreased the levels of DA and its turnover [134], depleted DA stores, and decreased DA release [135] in the striatum of rats, resulting in reduced dopaminergic neurotransmission, along with behavioral deficits, in rats [136]. In humans, the expression of a dopamine transporter was decreased in Mn-exposed patients [27], leading to the dysregulation of dopaminergic neurotransmissions [137]. We have previously reported that Mn induced dopaminergic neuronal injury and decreased the expression of tyrosine hydroxylase (TH) in the mouse brain [138,139,140]. Mn also increased the phosphorylation of TH at the serine 40 residue post-translationally and caused oxidative stress and apoptosis in PC12 cells [141]. We have found that Mn decreased TH mRNA and protein levels at the transcriptional level by decreasing the transcription factor repressor element-1 silencing transcription factor (REST) in dopaminergic cells [139]. The overexpression of REST increased its binding to the cis element of REST in the human TH promoter and interacted with histone acetyltransferase CREB-binding protein (CBP) to increase the TH expression [139]. REST also protected dopaminergic cells against Mn-induced neurotoxicity by enhancing its antioxidant and antiapoptotic proteins. The protective role of REST appears critical in Mn-induced dopaminergic neurotoxicity, as well as other neurodegenerative diseases, including PD [142] and AD [143]. Studies also showed that Mn impaired the TH activity via the activation of protein kinase Cδ and protein phosphatase 2A in dopaminergic cells [144]. These findings suggest that Mn impairs TH function by various mechanisms and promotes dopaminergic neuronal injury, leading to aberrant DA neurotransmission.

Glutamate. Mn dysregulates glutamate neurotransmission in the brain by impairing glutamate transporters and the N-methyl-D-aspartate (NMDA) receptor, leading to the overstimulation of postsynaptic glutamate receptors and excitotoxic neuronal death [145]. Studies have reported that Mn overexposure can lead to glutamate-induced excitotoxicity, as the inhibition of the NMDA receptor attenuated excitotoxic lesions in the striatum of Mn-exposed rats [146]. Mn impaired the NMDA receptor function by reducing the mRNA and protein levels of NMDA receptor subunits [147] and dysregulating phosphorylation of the NMDA receptor [148], suggesting that Mn modulate the NMDA receptor function at multiple levels involving transcription, translation, and posttranslational modifications. The Mn-induced dysregulation of the TrkB/Akt/Fyn-mediated phosphorylation of NMDA receptors was associated with the impairment of spatial memory and synaptic plasticity in mice [148]. In addition, Mn-induced glutamate dyshomeostasis could be associated with glutamate transporter dysfunction. We have previously reported that Mn decreased the glutamate uptake in astrocytes, along with a reduced expression of glutamate aspartate transporter 1 (GLAST) and glutamate transporter 1 (GLT-1), resulting in excess extracellular glutamate levels [8,149,150]. Since GLAST and GLT-1 are predominantly expressed in astrocytes and responsible for >90% of the glutamate uptake from the extracellular space in the brain [145], this Mn-reduced GLT-1/GLAST could directly contribute to excitotoxic neuronal injury. We have found that the Mn-decreased expression of GLT-1/GLAST was at the transcriptional level. The Mn-induced activation of the transcription factor Yin Yang 1 (YY1) was responsible for repressing GLAST and GLT-1 expression in both in vitro astrocyte cultures and in vivo mouse models [8,149,150]. Mn also modulated epigenetic modifier histone deacetylases (HDAC) to repress GLAST and GLT-1 expression by increasing the interaction of HDAC with YY1 and the subsequent histone deacetylation in astrocytes [149,150]. Astrocytic YY1 was critical in Mn-induced neurotoxicity, since astrocytic YY1 deletion attenuated the Mn-induced repression of GLAST and GLT-1 in the substantia nigra, concomitantly decreasing the interaction between YY1 and HDACs in astrocytes [8]. These findings indicate that Mn promotes neuronal injury by dysregulating the glutamate signal at least in part by the dysregulation of glutamate transporters GLT-1/GLAST and the NMDA receptor.

ACh. Although most relevant mechanisms for Mn neurotoxicity have been related to the brain dopaminergic systems [151], some features of manganism such as intellectual decline [152] cannot be explained by the disruption of brain dopaminergic systems alone. Mn effects on the cholinergic system may also contribute to the impairment in learning, memory, and locomotion [24]. Mn’s toxic effects have been suggested to be linked to an imbalance between the dopaminergic and cholinergic systems in the basal ganglia [153]. Studies have shown that Mn inhibited acetylcholinesterase (AChE), resulting in an increase in the production of ROS and reactive nitrogen species, leading to the intracellular accumulation of calcium in the rat brain [154]. Mn also decreased the activity of choline acetyltransferase (ChAT), thus reducing the production of ACh in striatal cholinergic terminals [155]. These Mn effects are more pronounced in developing brains, as the expression of ChAT is lower particularly in the midbrain and cerebellum of rats [156]. Mn inhibited the choline transporter, decreasing the choline uptake into cholinergic neurons in the hippocampus, cortex, and basal ganglia [157]. Mn also inhibited ACh binding to its receptors and decreased the expression of nicotinic acetylcholine receptors in the prefrontal cortex of mice, resulting in an impairment of spatial memory [158]. These various findings suggest that Mn could modulate the cholinergic system differently, depending on the experimental settings. 

GABA. Mn accumulates primarily in the globus pallidal GABAergic neurons of the basal ganglia, but the effects of Mn on GABAergic neurotransmission are inconsistent. Studies have shown that Mn dysregulated GABA neurotransmission by reducing GABA levels, leading to increased susceptibility to seizures in rats [25]. Mn also inhibited GABA uptake in the rat forebrain [159] and decreased GABA transporter 1 expression in the substantia nigra of rats, resulting in an increase of extracellular GABA concentrations [160]. In humans, Mn-exposed smelters showed that GABA levels were significantly increased in the thalamus and adjacent brain regions, but globus pallidus, which had the most Mn deposition, did not exert the highest alteration of GABA levels [161], suggesting that Mn neurotoxicity may result from the intrinsic vulnerability of neuronal systems to injury rather than the accumulated Mn levels in those local regions of the brain [162]. These findings indicate that Mn effects on GABAergic systems also vary depending on the experimental settings and conditions, requiring further studies to better understand how Mn modulates GABA neurotransmission.

### 3.4. αSyn Aggregation

αSyn, encoded by *SNCA,* is a major aggregate of Lewy body deposits in the brain of PD patients, and mutations in the *SNCA* are directly linked to the onset of PD [163]. αSyn appears to confer both neurotoxic and neuroprotective effects in PD experimental models, indicating that the role of αSyn in PD needs to be carefully interpreted. 

Studies have shown that the overexpression of αSyn exerted protective effects in dopaminergic cells against Mn-induced neurotoxicity in the short time of Mn exposure, while prolonged Mn exposure abolished αSyn’s neuroprotection against Mn toxicity and promoted αSyn aggregation [94], suggesting that the pathogenic mechanism of Mn involves αSyn misfolding. In *C. elegans* with mutations in *pdr1* and *djr1.1*, homologs of parkin and DJ-1 in humans, the overexpression of αSyn also attenuated Mn accumulation and oxidative stress [164]. 

Growing evidence also indicates the role of αSyn in Mn neurotoxicity, as Mn increased αSyn expression, aggregation, and subsequent cytotoxicity in different experimental models [86,165,166,167]. αSyn is involved in Mn-induced neurotoxicity by αSyn oligomerization and impaired autophagy in mice [168]. Mn increased the expression and aggregation of αSyn, leading to apoptosis in neuronal cells [28], while the knockdown of αSyn with antisense αSyn treatment [169] or siRNA [28] attenuated Mn-induced cytotoxicity, indicating that αSyn was critically involved in Mn-induced cytotoxicity. Mn induced conformational changes and the fibril formation of αSyn, leading to αSyn aggregation in an αSyn peptide solution [170]. Studies also revealed that Mn modulated other mechanisms such as the secretion and vesicular trafficking of αSyn between cells by increasing levels of RAB27A for the packaging of αSyn into exosomes [171], which, in turn, leads to the release of exosomes into the extracellular environment [172]. Serum samples collected from welders chronically exposed to Mn showed increased levels of misfolded αSyn in their exosomes, which have been shown to induce neuroinflammation and subsequent neurodegeneration [173].

### 3.5. Aβ Aggregation

Chronic exposure to high levels of Mn also serves as a risk factor in AD, as elevated levels of Mn were found in the parietal cortex and other regions of AD patients’ brains and Aβ aggregates in those regions and cognitive impairment [174,175]. Mn also induced the hyperphosphorylation of tau, another hallmark of AD, in PC12 neuronal cells [175]. Several mechanisms of Mn-induced toxicity contribute to the formation of Aβ plaques or amyloidogenesis in in vitro and in vivo AD models. Studies have shown that Mn increased the expression of Aβ precursor-like protein 1 (APLP1) and the formation of Aβ plaques in the frontal cortex of nonhuman primates [176,177]. Mn induced Aβ plaque formation and increased Aβ mRNA levels, as well as Aβ protein levels, by increasing β-secretase (BACE) and γ-secretase cleavage activities in the cerebral cortex and hippocampus of an AD mouse model, particularly in the microglia [178], suggesting that Mn-induced microglial inflammation likely contributes to amyloidogenesis and AD pathology. Mn could also increase the aggregation of misfolded Aβ peptides in cholinergic neurons via the dysregulation of proteasome 20S and heat shock proteins [179]. These findings indicate that Mn may contribute to AD pathogenesis via the Mn-induced dysregulation of Aβ production.

## 4. Concluding Remarks

Mn is an essential trace element required by various physiological processes in the body; however, chronic exposure to elevated Mn levels induces neurological disorders. Metal transporters are important in regulating Mn transport to maintain physiological levels of Mn in the body. The role of several Mn transporters was discussed to provide a better understanding of how Mn accumulates in the brain regions where it causes the most neuronal injury. Additionally, several aspects of the mechanisms of Mn-induced neurotoxicity, such as oxidative stress, inflammation, the dysregulation of neurotransmission, and αSyn aggregation, were reviewed. However, further investigations are needed to delineate the complex nature of the mechanisms of Mn-induced neurotoxicity, which could lead to identifying molecular targets in treating Mn toxicity, as well as other neurodegenerative diseases sharing common pathological features.

## Figures and Tables

**Table 1:** The molecular mechanisms involved in manganese (Mn)-induced toxicity in various neural cell types such as microglia, astrocytes, and neurons and in multiple regions of the brain.

Experimental Model	Species	Mn (Dosage/Concentration)	Cytotoxicity Mechanisms	Disease Model	References
*Neural cell type*					
Microglia	Mouse (N9), Mouse (BV2)	MnCl_2_ (50–1000 µM)	↑TNF-α, ↑ILs, ↑interferons ↑NF-κB activation	Mn toxicity	[93,125,128]
Microglia	Mouse (N9), Rat	MnCl_2_ (30 µM)	↑TNF-α, ↑IL-1β	Mn toxicity	[125,127]
Microglia	Human (HMC3)	MnCl_2_ (250 µM)	↑LRRK2 expression and kinase activity ↑ROS, ↑TNF-α, ↑apoptosis	Mn toxicity	[131]
Astrocytes	Mice	MnCl_2_ (0.1–10 µM)	↑Cyclooxygenase 2, ↑iNOS, ↑NF-κB activation, ↑inflammation	Mn toxicity	[118,120,121,122]
Astrocytes	Mouse	MnCl_2_ (50 µM)	↑Inflammation, ↑iNOS, ↑NO	Mn toxicity	[123]
Astrocytes	Rat, Mice	MnCl_2_ (5, 50 µM)	↑TNF-α, ↑IL-1β, ↑iNOS	Mn toxicity	[20,118,124,125,126]
Astrocytes	Rat, Human	MnCl_2_ (250 µM)	↓Glutamate reuptake, ↓EAAT1/GLAST, ↓EAAT2/GLT-1, ↑YY1, ↑HDAC-YY1 interaction	Mn toxicity	[149,150]
Astrocytes	Rat	MnCl_2_ (100–200 µM)	↓Glutamate reuptake, ↓SOD activity, ↓GPx activity, ↑LDH release, ↑IL-6	Mn toxicity	[99]
Astrocytes	Rat	MnCl_2_ (100, 500, 1000 µM)	↑F2-isoprostanes, ↑lipid peroxidation	Mn toxicity	[107]
Astrocytes	Human (U87)	MnCl_2_ (400, 800, 2000 µM)	↓Glutathione	Mn toxicity	[100]
*Neurons*					
Cortical Neuron	rat	MnCl_2_ (500 µM)	↑ROS production, ↑F2-isoprostanes, ↓ATP production	Mn toxicity	[22]
Dopaminergic	Human (SH-SY5Y)	MnCl_2_ (800 µM)	↑ROS production, ↑lipid peroxidation, ↓ATP levels, ↓mitochondrial membrane potential	Mn toxicity; PD	[101]
Dopaminergic	Human (SH-SY5Y)	MnCl_2_ (2–125 µM)	↑DNA damage, ↑oxidative stress	Mn toxicity; PD	[23]
Dopaminergic	Rat (N27)	MnCl_2_ (300 µM)	↑Cytochrome c release, ↑caspase-3 activation, ↑DNA damage, ↑ROS, ↑apoptosis, ↑αSyn aggregation	Mn toxicity	[94]
Dopaminergic	Rat (N27)	MnCl_2_ (10–5000 µM)	↑ROS, ↑mitochondria damage, ↑apoptosis, ↑PKCδ activation	Mn toxicity	[95,96]
Catecholaminergic/Dopaminergic	Mouse (CAD), Human (LUHMES)	MnCl_2_ (250 µM)	↓TH, ↓REST, ↓NRF2, ↓catalase, ↓HO-1 ↑oxidative stress, ↑inflammation	Mn toxicity; PD	[8]
Dopaminergic	Rat (PC12)	MnCl_2_ (100 µM)	↑TH phosphorylation, ↑cytotoxicity	Mn toxicity; PD	[141]
Dopaminergic	*C. elegans*	MnCl_2_ (40 mM)	↑oxidative stress	Mn toxicity; PD	[98]
Cholinergic	Mouse (SN56)	MnCl_2_ (1–200 µM)	↑Aβ peptides misfolding and aggregation	AD	[179]
Neuron	Mouse, Rat	MnCl_2_ (100, 500, 1000 µM)	↑F2-isoprostanes, ↑lipid peroxidation	Mn toxicity	[106]
Neuron	Rat (PC12)	MnCl_2_ (100–500 µM)	↑Tau phosphorylation, ↑impaired tau degradation/aggregation, ↑ERK/GSK-3β activation, ↑cytotoxicity	AD	[180]
Dopaminergic	Rat (MES 23.5)	MnCl_2_ (200–600 µM)	↑αSyn levels, ↑p38 activation, ↑NF-κB activation, ↑NO, ↑apoptosis	Mn toxicity; PD	[167]
Organotypic Brain Slices	Rat	MnCl_2_ (400 µM)	↑ROS, ↑apoptosis, ↑LDH release, ↓SOD activity, ↑αSyn levels	Mn toxicity; PD	[166]
Dopaminergic	Human (SH-SY5Y)	MnCl_2_ (500 µM)	↑Apoptosis, ↑caspase 3, ↑αSyn levels	Mn toxicity; PD	[169]
Dopaminergic	Mouse (MN9D)	MnCl_2_ (300 μM)	↑Exosome release, ↑cytotoxicity, ↑Rab27a	Mn toxicity	[172]
Dopaminergic	Human (LUHMES), Mouse (MN9D)	MnCl_2_ (10 mM)	↑αSyn misfolding, ↑αSyn release, ↑inflammation	Mn toxicity	[173]
*Brain regions*					
Striatum, brain stem	Rat	MnCl_2_ (25 mg/kg)	↓Glutathione synthesis, ↓energy ↑hypoxanthine, ↑xanthine, ↑uric acid		[102]
Basal ganglia	Human	NA	↑Astrogliosis	Mn toxicity; PD	[117]
Basal ganglia (Striatum); Substantia nigra	Rat (neonate, adult)	MnCl_2_ (10 mg/kg)	↓DA levels	Mn toxicity; PD	[132,133]
Striatum	Rat	Mn (100 mg/kg)	↓DA levels and stores, ↓DA turnover, ↓DA release, ↑behavioral deficits	Mn toxicity; PD	[134,135,136]
Globus pallidus (Striatum), Midbrain	Human	NA	↑Mn levels, ↓DA transporter	Mn toxicity	[111]
Striatum	Rat	MnCl_2_ (5–20 mg/kg, daily, 20 d)	↑Motor deficits, ↑oxidative stress	Mn toxicity; PD	[21]
Striatum	Rat	MnCl_2_ (0.5–2 µmol/µL)	↑Excitotoxic lesions; ↑oxidative stress, ↑mitochondrial impairment	Mn toxicity	[146]
Striatum	Rat	MnCl_2_ (1 mg/mL)	↓ChAT, ↓ACh levels,	Mn toxicity	[155]
Striatum	Mouse	MnCl_2_ (50, 100, and 200 μmol/kg)	↑Autophagy dysregulation, ↑cell injury. ↑αSyn	Mn toxicity; PD	[168]
Hippocampus	Mouse	MnCl_2_ (100 µmol/kg, 5×/week, 6 weeks)	↑Impaired NMDA phosphorylation, ↑memory dysfunction, ↑αSyn dysregulation	Mn toxicity; AD	[148]
Whole brain	Rat	MnCl_2_ (30 mg/kg)	↓AChE, ↑oxidative and nitrosative stress, ↑intracellular Ca^2+^ accumulation	Mn toxicity	[154]
Whole brain	Rat	MnCl_2_ (3 mg/mL)	↓GABA, ↑seizure duration, ↓seizure threshold	Mn toxicity	[25]
Forebrain	Rat	MnCl_2_ (10 mM)	↓GABA uptake	Mn toxicity	[159]
Thalamus	Rat	MnCl_2_ (1 g/L)	↑GABA	Mn toxicity	[160]
Substantia nigra	Mouse	MnCl_2_ (30 mg/kg, daily/3 wks)	↓TH, ↓EAAT1/GLAST, ↓EAAT2/GLT-1, ↑YY1, ↑HDAC-YY1 interaction, ↑motor deficits, ↑microglial activation	Mn toxicity; PD	[8]
Midbrain, cerebellum	Mouse	MnCl_2_ (10 mg/mL)	↓ChAT, ↑impaired choline uptake	Mn toxicity; encephalopathy	[156]
Hippocampus, cortex, and basal ganglia	Rat	MnCl_2_	↓Choline transporter, ↓choline uptake, ↓homovanillic acid	Mn toxicity	[157]
Prefrontal cortex	Mouse	MnCl_2_ (5 mg/kg, daily/22 d)	↓ACh activity, ↓nicotinic ACh receptors, ↑impaired spatial memory	Mn toxicity; AD	[158]
Frontal Cortex	Nonhuman primate	Mn (3.3–5.0, 5.0–6.7, 8.3–10 mg Mn/kg)	↑αSyn aggregation, ↑apoptosis	Mn toxicity; PD	[165]
Frontal cortex	Nonhuman primate	NA	↑Aβ precursor protein 1, ↑Aβ expression, ↑cytotoxicity	AD	[176,177]
Parietal cortex	Human	NA	↑Mn levels, ↑AD pathology, ↑impaired Aβ and ApoE function	AD	[174]
Cerebral cortex, hippocampus	Mouse	MnCl_2_ (0.36 mg/mL, 5 mos.)	↑Aβ expression, ↑β- and γ-secretase cleavage activities	AD	[178]
Blood	Human	NA	↑Mn levels, ↑impaired Aβ degradation/aggregation, ↑cognitive impairment	AD	[175]

↑: increased. ↓: decreased. NA, not applicable/not available. CAD: Cath.a differentiated. LRRK2: leucine rich repeat kinase 2. LUHMES: Lund human mesencephalic. TNF-α: tumor necrosis factor alpha. NF-κB: nuclear factor kappa-light-chain enhancer of B cells. IL-1β: interleukin 1β. IL-6: interleukin 6. iNOS: inducible nitric oxide synthase. NO: nitric oxide SOD: superoxide dismutase. GPx: glutathione peroxidase. LDH: lactate dehydrogenase. ROS: reactive oxygen species. EAAT1/2: excitatory amino acid transporter 1/2. GLAST: glutamate aspartate transporter 1. GLT: glutamate transporter 1 PKC: protein kinase C. GSH: glutathione. NRF2: Nuclear factor erythroid 2-related factor 2. HO-1: Heme oxygenase-1. REST: repressor element-1 silencing transcription factor. TH: tyrosine hydroxylase. DA: dopamine. αSyn: α-synuclein. Aβ: amyloid-β. ApoE: apolipoprotein E. ChAT: choline acetyltransferase. ACh: acetylcholine. GABA: gamma aminobutyric acid. ERK: extracellular signal-regulated kinase: GSK-3β: HDAC: histone deacetylase. YY1: Yin Yang 1. NMDA: N-methyl D-aspartate. PD: Parkinson’s disease. AD: Alzheimer’s disease.
